# Current State of Safe Pregnancy Policies for the US Surgical Trainee

**DOI:** 10.1002/oto2.172

**Published:** 2024-07-21

**Authors:** Hayley Mann, Tiffany Glazer

**Affiliations:** ^1^ Department of Surgery, Division of Otolaryngology–Head and Neck Surgery University of Wisconsin Hospital and Clinics Madison Wisconsin USA

**Keywords:** pregnancy, pregnant surgeons, resident safety, trainees

## Abstract

**Objective:**

Define current practices and protocols in surgical training programs for pregnant trainees.

**Study Design:**

Cross sectional.

**Setting:**

Academic surgical training programs in the United States.

**Methods:**

A validated 20‐question survey was sent via email to program directors and coordinators of US surgical training programs, including otolaryngology head & neck surgery (OHNS), plastic surgery, vascular surgery, and general surgery. The survey was issued in November and December 2022 and data were collected until January 2023. This study was approved for exemption by the Minimal Risk Research IRB at the University of Wisconsin Madison (ID number 2022‐1370).

**Results:**

Surveys were emailed to 608 surgical programs, and the response rate was 23.5% (143/608) including 45 OHNS programs. When asked if their program has a policy in place for pregnant trainees, 84.4% responded yes, and 82.4% responded that they were satisfied with their policy. Subsequent questions addressed individual policies and risk factors facing pregnant trainees. 60.3% of programs report providing protected time off for miscarriages. Only 36.9% provide information to pregnant trainees regarding workplace exposures that pose a risk of fetal anomaly or miscarriage. Only 47.1% incorporate rest breaks for pregnant trainees, and only 20% protect the number of hours a pregnant trainee operates per week. 24.2% adjust overnight shifts or call schedules for pregnant trainees, and of those that adjust call shifts, 20% require pregnant trainees to “make up” these missed call shifts. Less than half (40%) of programs have a contingency plan in place for supporting nonchild‐bearing residents who may take on the work of their colleagues during pregnancy or postpartum.

**Conclusion:**

While a majority of training programs report a pregnancy policy in place for trainees, most of these policies appear to be severely deficient in addressing critical aspects of surgical training that place both fetus and mother at significant risk of complications. This data indicates a need for a safe pregnancy protocol in order to protect both surgeon and fetus.

According to 2017 AAMC data, less than 25% of practicing US surgeons are female.^1^ However, beginning in 2019 for the first time in history, women now make up >50% of incoming US medical students. The number of female medical students choosing a surgical specialty is also increasing, although slowly and disproportionately—females now make up 36.2% of US surgical residents in the 2021 to 2022 academic year.^2^ Nineteen percent of practicing otolaryngologists identify as female, with a significantly higher proportion of women in earlier practice years, suggesting progress within this field to close the gender gap.^3^ With this welcome change toward achieving gender parity in surgery brings to the forefront a challenge that is uniquely female—that of childbearing.

Barriers to pregnancy are unfortunately pervasive throughout all medical specialties, given that most female residents are in their peak reproductive years during residency, fellowship, and early career as faculty.^4^, ^5^ Childbearing challenges particularly impact female trainees in surgical specialties given the rigor of training, length of training, and culture. Consequently, many female medical students are dissuaded from pursuing a surgical residency due to family planning and work‐life balance concerns.^6^, ^7^ In a survey sent to women in otolaryngology in 2018, 83% felt it is “somewhat to very difficult” to manage work‐life balance, and 48% to 98% of female surgical residents voluntarily delay childbearing.^8^ Surveys of female residents have cited multiple reasons for this decision, including: (1) the desire to avoid disruption or prolongation of training; (2) burdening others with call or shift coverage; (3) the concern that having children will negatively impact one's future career; (4) the negative stigma attached to being a pregnant surgeon; (5) the fear of obstetric complications secondary to the high stress and intense workload of surgical training; (6) work‐life balance concerns; (7) the lack of a formal policy or support from their program to protect them; (8) inadequate time off; (9) obstacles towards breastfeeding; and (10) personal factors that affect family planning such as relationship status, age, and finances.^6^, ^7^, ^8^, ^9^, ^10^, ^11^, ^12^ One study demonstrated that of females who had a child during residency, 40% strongly considered leaving their career, and nearly 30% would discourage female medical students from entering a surgical career, specifically because of the difficulties of balancing pregnancy and motherhood with training.^11^

The challenges that female surgeons face with childbearing are unique, specifically when compared to their male surgeon and female nonsurgeon colleagues. Compared to their male counterparts, female surgical residents are significantly more likely to delay childbearing due to surgical training, have fewer biological children, and are more likely to require the use of assistive reproductive technology (ART).^12^ Sixty‐one percent of female surgical residents (compared to 16% of male residents) felt that having a child in residency would be viewed unfavorably by program leadership.^13^ In one study, 35% of otolaryngology program directors felt there was a strong negative effect of surgical trainee pregnancy on co‐residents and expressed concerns regarding education, service coverage, and hospital costs.^14^ Female surgical residents are more likely than nonsurgeon female partners to have major pregnancy complications, and this finding persisted after controlling for age, work hours, race and ethnicity, and use of ART.^15^ Female surgical residents are also more likely to undergo nonelective cesarean sections and experience postpartum depression compared to their nonsurgery female colleagues.^16^

Despite these unique risks and ongoing gender disproportionality in otolaryngology, there are few studies demonstrating clear institutional or program‐specific policies that address the safety of pregnant surgical residents. The ACS along with the ACGME Residency Review Committee developed a statement in August 2020 which described the following for protection of pregnant residents: the residency program is responsible for supporting the medical needs of the trainee; the residency program should create a schedule that is flexible and equitable for the trainee to take leave, while accounting for those health care professionals who are affected by their absence; and the residency should provide at least six weeks of paid parental leave for either or both parents.^17^

As women comprise an increasingly larger proportion of surgical trainee positions, careful and deliberate consideration of policies that promote a safe pregnancy in this population is a necessity for any program. This study sought to collect data from surgical training programs regarding their current policies and codified protections in place for pregnant trainees.

## Methods

In order to understand the current perinatal policies of US surgical residencies as it relates to their pregnant trainees, we created a 20‐question survey that was reviewed and validated by expert consensus (Table 1). The survey questions were developed by performing cognitive interviews with 2 members of a perinatal policy workgroup with particular attention paid to the resulting information and inferences the survey would yield to provide validity to the results.^18^ This was followed by a pilot survey sent to 2 Department of Surgery faculty who were not involved in the workgroup and their feedback was incorporated. This survey was sent via email to program directors and coordinators of US surgical training programs, including otolaryngology head & neck surgery (OHNS), plastic surgery, vascular surgery, and general surgery. Program director and coordinator emails were identified through the ACGME website catalog of programs and associated contact information. The survey was issued in November and December 2022 and data were collected until January 2023. This study was approved for exemption by the Minimal Risk Research IRB at the University of Wisconsin Madison (ID number 2022‐1370). To promote disclosure and accuracy of survey data, results remained de‐identified in all categories, including surgical subspecialty, program size, demographic composition, and region. Anonymous responses were compiled and subsequently subjected to descriptive analysis.

**Table 1:** Pregnancy Policy Survey Sent to 143 Surgical Training Programs

Safe Pregnancy Survey Questions
What is your role?
What type of training program are you involved in?
How many residents does your program have per PGY class?
Does your program currently have any female trainees?
Does your program currently have any pregnant female trainees?
Has your program ever had pregnant female trainees?
Does your program have a policy or accommodations in place for pregnant trainees?
Would you like to have accommodation guidelines for pregnant trainees?
Are you satisfied with the policy or accommodations for pregnant trainees?
Does your program allow flexible hours for pregnant trainees to attend prenatal or fertility appointments?
Is there any protected time off for bereavement/miscarriage in your program?
Do you provide information to pregnant trainees regarding workplace exposures that pose a risk of fetal anomaly or miscarriage?
Does your program incorporate rest breaks for pregnant trainees?
Are stools or chairs available for pregnant trainees in the clinic and OR?
Does your program protect the number of hours a pregnant trainee operates per week?
Are overnight shifts or call schedules adjusted for pregnant trainees?
Does your program require a pregnant trainee to make up these overnight shift or call activities?
Does your program have a contingency plan for supporting non‐child‐bearing trainees if their pregnant colleagues’ responsibilities are adjusted during pregnancy or post‐partum?
Any additional comments?

## Results

**Figure 1:**
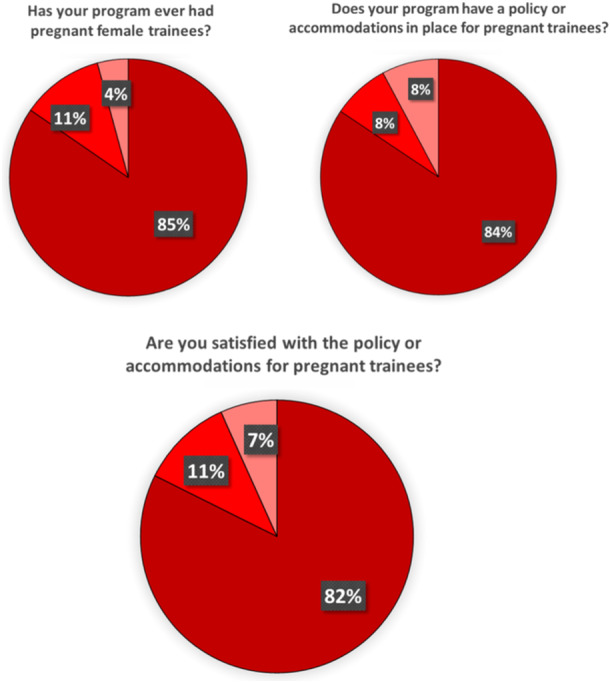
Survey data collected from 143 US surgical training programs: accommodations in place.

Surveys were emailed to program directors and coordinators in 608 surgical programs, and the response rate was 23.5% (143/608). Most responses were from general surgery (70), followed by OHNS (45), plastic surgery (20), and vascular surgery (8). Over 84% of programs have had a pregnant trainee in the program at some point (Figure 1).

**Figure 2:**
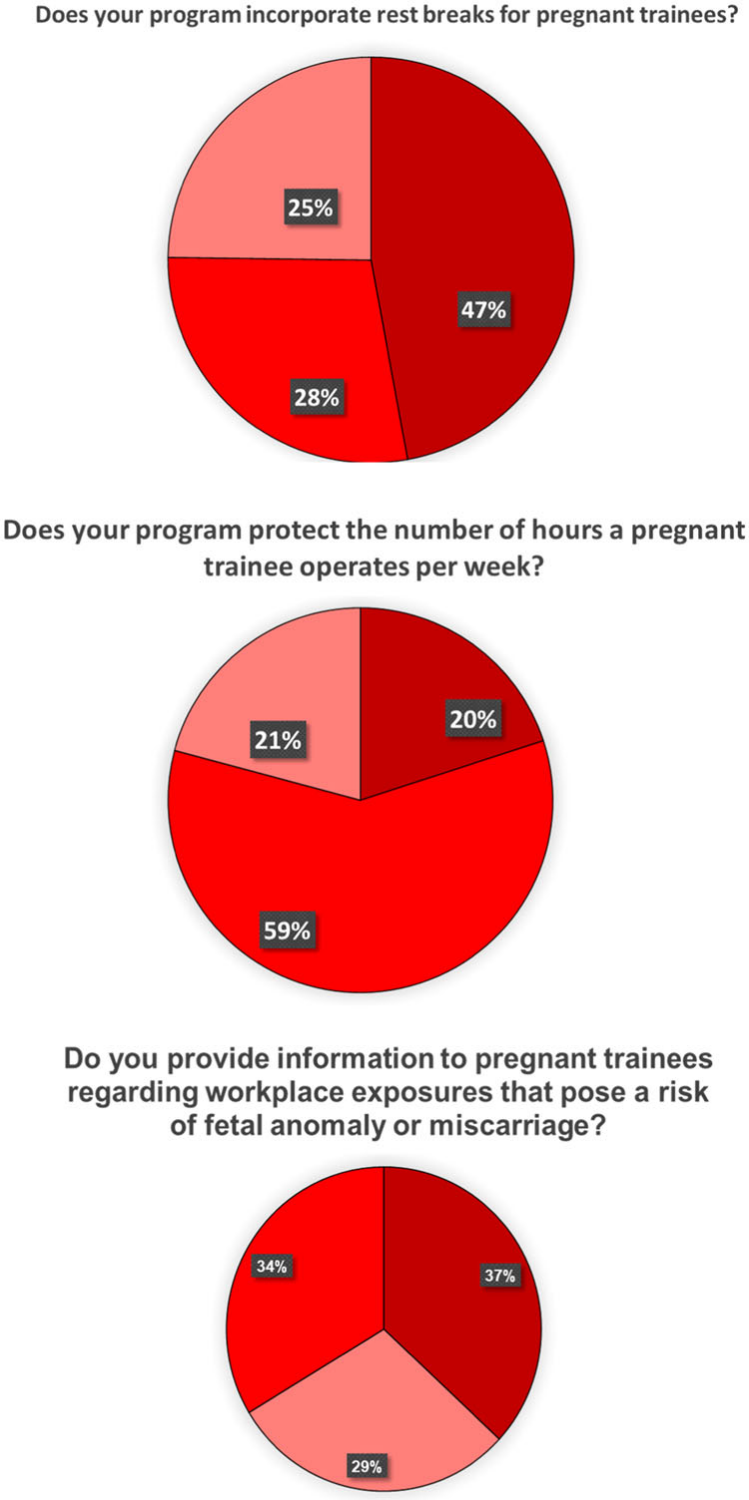
Pregnancy safety policies currently in place at 143 US surgical training programs.

**Figure 3:**
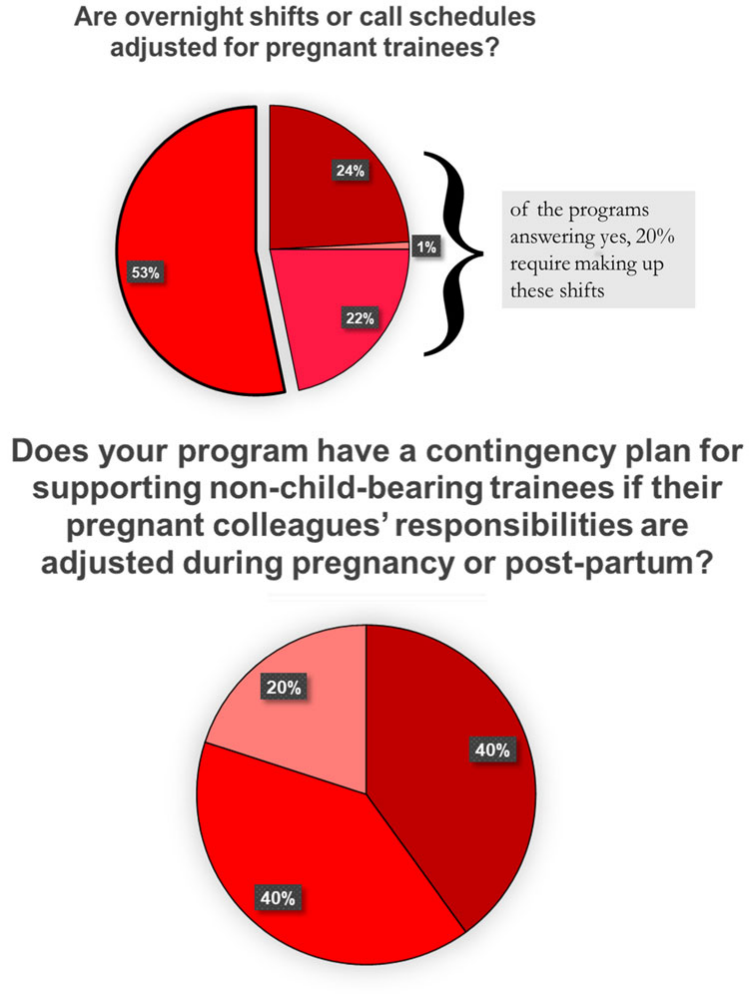
Policies in place regarding call protections for pregnant surgical trainees.

**Figure 4:**
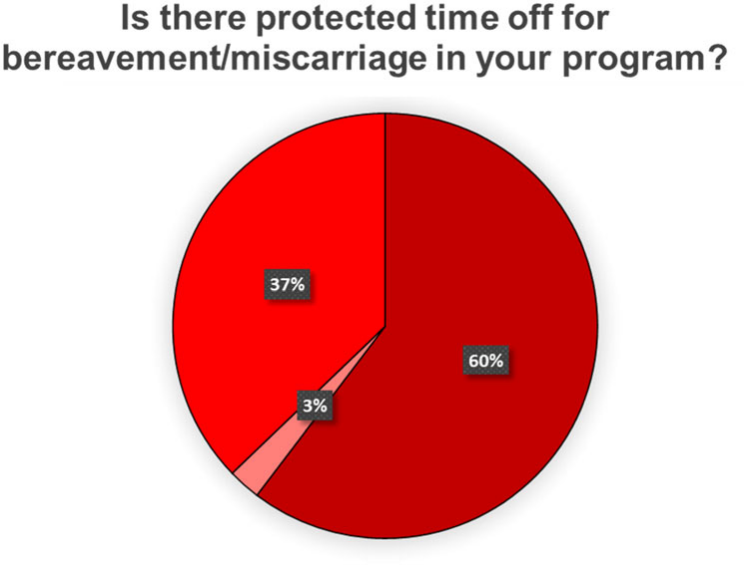
Protected time for bereavement as it regards to pregnancy/pregnancy loss in US surgical training programs.

When asked if their program has a policy in place for pregnant trainees, 84.4% affirmed their program has a policy in place for pregnant trainees (Figure 1), and 82.4% responded that they were satisfied with their policy (Figure 1). 47.1% incorporate rest breaks for pregnant trainees (Figure 2), and 20% protect the number of hours a pregnant trainee operates per week (Figure 2). 45.8% adjust overnight shifts or call schedules for pregnant trainees, with 21.7% of them adjusting schedules during the third trimester only (Figure 3). Of those programs that adjust call shifts, 20% require pregnant trainees to “make up” these missed call shifts (Figure 3). Flexible scheduling for fertility and prenatal appointments was provided for all residents at 76.4% of responding programs, while 8% only provided this for female residents and 13.8% of programs were unsure of if there was a relevant policy in place. 60.3% of responding programs give protected bereavement time for miscarriages (Figure 4), with 3 programs responding that this is only provided for female residents and 29 programs responding that they did not know if such a policy was in place at their institution. 36.9% provide information to pregnant trainees regarding workplace exposures that pose a risk of fetal anomaly or miscarriage, such as ionizing radiation or anesthetic gas exposure (Figure 2). Less than half (40%) of programs have a contingency plan in place for supporting nonchild‐bearing residents who may take on the work of their colleagues during pregnancy or postpartum (Figure 3).

Respondents were allowed to leave additional comments after completing the survey; all responses received are shown (Table 2).

**Table 2:** Additional Free‐Text, Optional Survey Responses From 143 Surgical Training Programs Regarding Perinatal Policies for Residency Programs

Optional additional comment responses
**Resident‐led informal policies**
Many of these things are in place but not formally—we have had many pregnant trainees and the culture is supportive but does not mandate rest breaks, etc. Pregnant trainees are not required to make up call but they do because they personally do not want to burden their co‐residents, this survey makes me realize we should consider formalizing that in our program.
Residents have a big say in adjusting schedule to accommodate births and parental leave. Administration is consulted for policy and schedules are built around due dates by our Admin chief.
Training accommodations are made as needed by individual circumstance and not by blanket policy. Discussed with residents.
In the past we have been accommodating with any pregnancy/birth related residents. Although items may not be officially in policies, our residents do usually come together and help each other out when needed.
We are a small program. We do whatever we need to to support the resident. The five‐in‐six ABS option has been used by two residents, one who used it to have two children in three years and to take reasonable maternity leave around the births.
All the questions on the previous page can be answered with the flexibility we allow our residents in general, to go to the doctor for any reason and to have family time as needed. They support each other and adjust call as needed.
**Difficulties due to program size**
Very difficult for small programs to accommodate this. Included in this should be support for APP's provided by the institution to help accommodate pregnant trainees.
Our program is alternating 1‐2‐1‐2‐1 as far as the residency composition. Our workforce is small, and in a lot of ways, it's nearly pushed to its limit. We have not yet had a pregnant resident, or a resident whose spouse was pregnant. I do believe it is important that we do allow for those who are pregnant accommodations, but also trainees who are not the one who is pregnant to be able to attend prenatal visits, etc. I think your question about the non‐pregnant trainees who are involved is a very interesting one. I know that even with my residents who previously had taken time off to attend fellowship interviews in the past, with how that altered the call schedule in an already small call pool, the resident dynamic can become somewhat hostile.
**Call responsibilties**
We do not currently lessen call responsibility for pregnant residents, but we are actively reviewing our policy. Parental leave is available for all new parents regardless of gender, sex, or pregnancy status.
Currently the pregnant females take the same amount of call, OR time, clinic as non‐pregnant resident. If they choose to split any 24 hour call shifts they do two 12 hour shifts instead. If the pregnant resident has a night float rotation during the third trimester then we change the schedule so that they are on night float month earlier in their pregnancy. The current guidelines regarding pregnancy lack detail regarding reduced work hours. If a pregnant resident requests to work 40 hours per week for the final 4 months of their pregnancy should we consider that 50% clinical and extend their training by 2 months? I think that there should be clear guidelines on how to address reduced duty hours in terms of board eligibility. In my opinion, working/training “part time” should be a clearly defined accommodation option.
Wish we could offer long maternity leave, but understand the potential impact to training length. Maybe the EPAs will help this.
New Policy Formation/Initiation
This is timely because we are currently revamping our entire policy and I hope that all my “no” answers become “yes” answers shortly.
We just recently updated our birthing and non‐birthing parenting policy. I found a wide variety of attitudes both among residents and faculty. I had to institute a policy that was not universally well received, but felt it respected the health of mom and baby
You highlight many important points that are currently under consideration as we rewrite our policy regarding residents during pregnancy.
Guidelines are needed as to how to better support early pregnancy with call protection/work hour limitations—this is complicated given the need to know about early pregnancy to effect change and implement plans. I strongly believe that we have lost focus on the child bearing female with the all encompassing “Leave policies” that have been put forward and implemented over the past year ‐ they have been equated to any “parent” which completely minimizes the childbearing experience. I hope this group will put forward specific recommendations to help us improve the tragic outcomes of fetal loss and premature delivery. We want to support our female trainees in family planning ‐ we are hungry for data and recommendations to improve the course.
**No formal policy in place**
We may not have a specified plan for each of the situations in this survey, but we (as a faculty and resident group) are adaptable to look after and care for each other. When a situation arises in which we don't have specified policies, we deal with it in the best interest of the resident.
I feel we have a supportive and open program, and a majority of our residents have children during their residency. At times we have had more than one female resident pregnant that created a significant strain on the program.
Most arrangements for pregnant residents are done at the team level and formal plans are then submitted to the Program Director for documentation.
Our program director and chief resident along with faculty work on plans of coverage based off cases that come up with residents. It could be pregnancy, resident caregiver, deaths, etc.
Most of these questions revolve around clinic responsibilities. Therefore, these are recognized in the clinical setting and acknowledged as needed. Additionally, we follow the ABO‐HNS and the institution's policies. We accommodate all residents as needed.
No official policies on the books but we are very sensitive to risks of miscarriage and preterm labor and both co‐residents and attendings try to be proactive in their support of rest and fewer hours during the 3rd trimester in particular.
I just gave birth 2 weeks ago. While my program was supportive there were no standardized protocols in place and no adjustments were made to my schedule.
Apologies. I have only been in my role for one year, so I have not had to address any pregnant trainees workload/benefits/accommodations.

## Discussion

The development of clear and translatable policies surrounding perinatal care of surgical trainees is essential for attracting and retaining talented surgical trainees and faculty, protecting surgeon mothers and their unborn children, and decreasing the burden felt by nonchildbearing colleagues. The policy must also balance providing exceptional patient care and adequate surgical training. In our study, while over 80% of programs stated they have a policy in place to protect pregnant trainees, recommended accommodations and protections, such as adjusting overnight shifts or incorporating rest breaks in long OR cases, were either not present or unknown to be present in many of these policies. One way to interpret this data is that many programs conflate perinatal policies (ie, policies that protect a surgeon while pregnant) with parental policies (ie, parental leave). The vast majority of parental policies address parental leave in the postpartum period, with very few addressing the specific risks that trainees face *during* pregnancy, including call requirements, environmental risks, and physical risks.

Female surgical residents face numerous obstacles that result in delays and complications in starting a family.^19^ The demands unique to the job, fears, and stigma associated with being a pregnant woman in surgery can come at the significant future cost of increased infertility and maternal complications. The majority of female residents overestimate the age of fertility decline and misjudge the time available to have children.^20^ One‐third of female physicians with infertility said they would have attempted conception earlier if they had the chance to do it again.^21^

Infertility in female surgeons is a well‐documented phenomenon and cements the need for protection of trainees who are largely in their prime childbearing years. Female surgery resident infertility rates are as high as 30%–32% compared to 10.9% in the general population, and 84% of these trainees require an infertility workup. In approximately 33% of residents, the cause of infertility could not be determined.^22^, ^23^ Potential reasons in these cases may be secondary to the physical and psychological demands of surgical residency, decreased frequency of intercourse, or the fact that female surgery trainees are more likely to be single or divorced than their male counterparts. Approximately 18%–28% of female surgical trainees seek fertility support with assisted reproductive technology (ART), a rate significantly higher than in the general population (5.2%–12%)^23^, ^24^ A formalized institutional policy could help mitigate the cited concerns surgical trainees have in becoming pregnant during residency and therefore decrease the risk of infertility related to the delay of pregnancy.

Those female residents who are able to conceive are then faced with a significantly higher risk of obstetric complications. Average obstetric complication rates for female surgery trainees range from 25% to 82% in the literature and are considerably higher than the general US population at 5%–15%.^9^, ^15^, ^25^ Furthermore, 42% of female surgeons report at least one pregnancy loss, more than twice the national average. Of those who had a miscarriage, 1 survey reported that greater than 75% took no days off to grieve or recover physiologically and emotionally.^15^, ^26^ The complications with the highest incidence include involuntary miscarriage (13.3%), preterm delivery (PTD) (10.5%), and intrauterine growth restriction (10.5%). The need for early induction in female surgical trainees is also significantly higher than the average population at 31.8% compared to 21.7%.^24^

Increased obstetric complication rates have been associated with modifiable and intervenable risk factors such as total work hours, call duty, noise, standing, and operating hours, among others.^15^, ^25^, ^27^ Higher obstetric complication rates can be associated with working more than six call shifts per month (49% vs 26.4%) and more than 8 hours per day (41.7% vs 8.9%).^26^ One article by Hamilton et al found that the odds of experiencing PTD increased by greater than four‐fold in trainees who worked greater than 60 hours per week while pregnant.^23^ Other potential factors that can increase complication risk include prolonged standing, physically challenging work, and irregular hours such as night shifts.

Pregnant surgical trainees also face psychosocial challenges during pregnancy. While there are varying degrees of support at different programs, many pregnant female surgical trainees still face a pervasive negative perception of commitment to one's career by leadership, coined the “motherhood penalty.” Multiple survey studies have cited that 38% to 61% of program directors responded that becoming a parent negatively affects a female trainee's performance.^28^ Many carry the guilt and fear of being viewed as “a burden on colleagues” or “not pulling their weight” and are put into a position of choosing between their health, their fetus's health, and their work. In one survey study of over 200 female surgical residents, only 12% of respondents had decreased duty hours while pregnant and approximately 95% continued overnight call up until delivery.^11^, ^25^ In another report of women who gave birth while participating in a general surgery residency, 85% worked an unmodified schedule before delivery, and more than three‐fourths took less than 6 weeks of maternity leave.^29^ Many residents must find coverage during maternity leave or make up missed call once they return to clinical duty with a newborn at home. Some programs have suggested postpartum mothers should start making up call while still on maternity leave to ease the burden. Additional studies have demonstrated no difference in case log volume between female residents who did and did not have a child, again showing the desire to avoid being viewed as “lazy” during and after pregnancy.^30^

The Association of Women Surgeons suggests that pregnant residents take less demanding rotations in the first trimester and first postpartum month.^17^ One general surgery program has instituted a policy for a “safe and supportive” culture surrounding pregnant residents including eliminating overnight shift work; modifying home call to include either no home call, home call with back‐up available for inpatient assessments or operations, or reduced frequency of home call; and codifying the protection for pregnant surgical residents to leave the operating room for more frequent hydration, rest, and other bodily needs.^31^ This policy also addresses the needs of resident surgeons and their partners following the birth of their child by offering a 2‐week transition period following parental leave where residents can request no 24‐hour call, duty changes to provide more predictable working hours, and less physically strenuous rotations.^31^ Some non‐surgical residency programs have instituted opt‐out policies, reducing the burden of self‐advocacy on the pregnant resident. One emergency medicine program piloted one such policy change that included no nights or call during the first and third trimesters, a 6‐week new parent flexible scheduling policy, and clarified sick call options.^32^, ^33^ This pilot study, albeit a small cohort from a single institution, was not felt to increase work burden on the chief residents and was approved by majority of the nonpregnant residents.^32^, ^33^ These recommendations and policy changes work to protect the health and safety of pregnant residents and demonstrate the necessity of flexibility and individualization that allow these changes to work.

Our data is limited due to the survey nature of the study, response rate, recall bias, and respondees largely comprised of program leadership. Respondees may have conflated perinatal policies with parental leave policies. Our data does not necessarily reflect the lived experiences of pregnant trainees and is unable to fully characterize the codified policies at responding programs. Despite these limitations, our data gives insight into the current understanding of training programs on the necessity of protections for the pregnant trainee and the immense policy variation between programs.

While the ACS guidelines and others help point programs in the right direction regarding safe prenatal care for trainees, there is no universal mandate or regulation of any kind. Training programs are left to interpret and enforce guidelines themselves. Furthermore, trainees are vulnerable within the hierarchy of their training programs, and most pregnant trainees are unlikely to advocate for themselves despite the existence of such guidelines. Areas of future research include directing data collection at what percentage of pregnant surgeons utilize their program's policy and what factors increase their ability to do so. Additionally, objective measures regarding knowledge of potential safety and health‐related factors in the surgical workplace as it relates to pregnancy should be collected for both child‐bearing surgical residents, their colleagues, and faculty. It is incumbent on the training program to protect the pregnant surgeon, so she does not have to struggle to find her balance between maintaining personal safety and continuing work responsibilities. Our data suggests that policies in place at surgical training programs are highly varied and many do not provide protections for the unique challenges that pregnant surgical trainees face. Codified guidelines to protect the surgical resident are a necessity in order to promote and maintain diversity within Otolaryngology.

## Author Contributions

**Tiffany Glazer**, design, conduct, analysis, presentation of research; **Hayley Mann**, design, analysis, presentation of research.

## Disclosure

### Competing interests

None.

### Funding source

None.
